# Synchronization of the 12-h circatidal rhythm maintains the kidney through repeated cycles of warm reperfusion in the hibernating ground squirrel

**DOI:** 10.1152/function.015.2026

**Published:** 2026-06-26

**Authors:** Austin E. Gillen, David J. Orlicky, Rui Fu, Swati Jain, Alkesh Jani, Sandra L. Martin

**Affiliations:** ^1^RNA Bioscience Initiative, University of Colorado School of Medicine, Anschutz Medical Campus, Aurora, Colorado, United States; ^2^Department of Medicine, University of Colorado School of Medicine, Anschutz Medical Campus, Aurora, Colorado, United States; ^3^Department of Pathology, University of Colorado School of Medicine, Anschutz Medical Campus, Aurora, Colorado, United States; ^4^Department of Cell and Developmental Biology, University of Colorado School of Medicine, Anschutz Medical Campus, Aurora, Colorado, United States

**Keywords:** acute kidney injury, biological rhythm, chronic kidney disease, organ preservation, renal transplant

## Abstract

The hibernating 13-lined ground squirrel kidney is a unique natural model of resistance to damage caused by cold storage and warm reperfusion. Over months, the kidney is exposed to cycles between multiday periods of torpor with low perfusion at ice-cold temperature and rapid warm reperfusion during arousals. Serum creatinine accumulates during torpor but normalizes during arousal, and animals emerge each spring with functioning kidneys. After confirming a lack of kidney histopathology in sections from 11 animals that had completed 11–22 torpor-arousal cycles, we collected RNA sequencing (RNA-Seq) data from 32 ground squirrel kidneys representing 6 key transitional time points based on seasonal and torpor-arousal cycle physiology. Hibernation state-specific gene expression changes were identified after removing three informative outliers. Both seasonal and torpor-arousal cycle-specific gene expression changes were found. These differentially expressed genes illuminated molecular mechanisms that mitigate damage while supporting full recovery during each ∼12 h rewarming. As with the response to renal ischemia-reperfusion injury in other species, the arousing hibernator induced immediate early genes during warm reperfusion. But, in the hibernator, this response did not precipitate the gene expression program of maladaptive repair that is characterized by cell death, immune system activation, and fibrosis. Rather, it appears that induction of immediate early genes activated a universal 12-h, “circatidal” rhythm. The efficient unfolding of this rhythm across each arousal from torpor was facilitated by the seasonally changed background primed for rapid cell division and minimal energy consumption. Adaptive repair was achieved, with proteostasis and cell type-specific function restored, assuring that minor damage did not accumulate.

**NEW & NOTEWORTHY** The hibernator kidney exhibits no damage after 10–22 cycles between prolonged cold exposure and rapid warm reperfusion occurring over several months. As found with kidney damage in other species, each rewarming activates an immediate early gene response. Unique to the hibernator, adaptive repair is completed within 12 h, facilitated by rapid activation of the universal “circatidal” rhythm unfolding on a seasonally primed background that assures minimal cell death, rapid cell division, and repair.

## Graphical abstract

**Figure:**
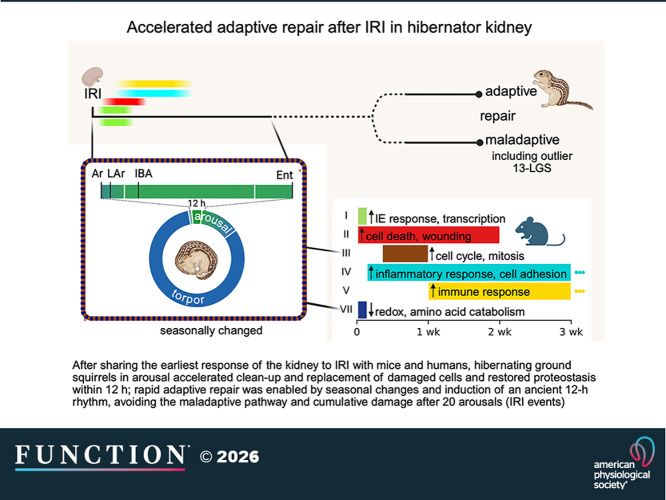

## INTRODUCTION

Acute kidney injury (AKI) describes the sudden loss of kidney function, typically characterized by increased serum creatinine and decreased urine output. AKI may either be reversed with kidney function restored or progress to chronic kidney disease (CKD) and renal failure. The chance of functional reversal decreases with increasing intensity and occurrence of injury, i.e., increasing frequency, duration, or severity of insult (1, 2). Common examples in humans and animal models include increasing time after decrease or cessation of blood flow in ischemia-reperfusion injury (IRI) (3, 4), toxin exposure (5), or, in the case of renal transplant, cold storage time (6). Increasing frequencies of AKI, the large burden of chronic kidney disease (7), and the unmet need for transplantable kidneys make it crucial to devise new strategies to prevent the progression of AKI to CKD, as well as to improve graft survival during kidney storage and transplant.

**Figure 1.**
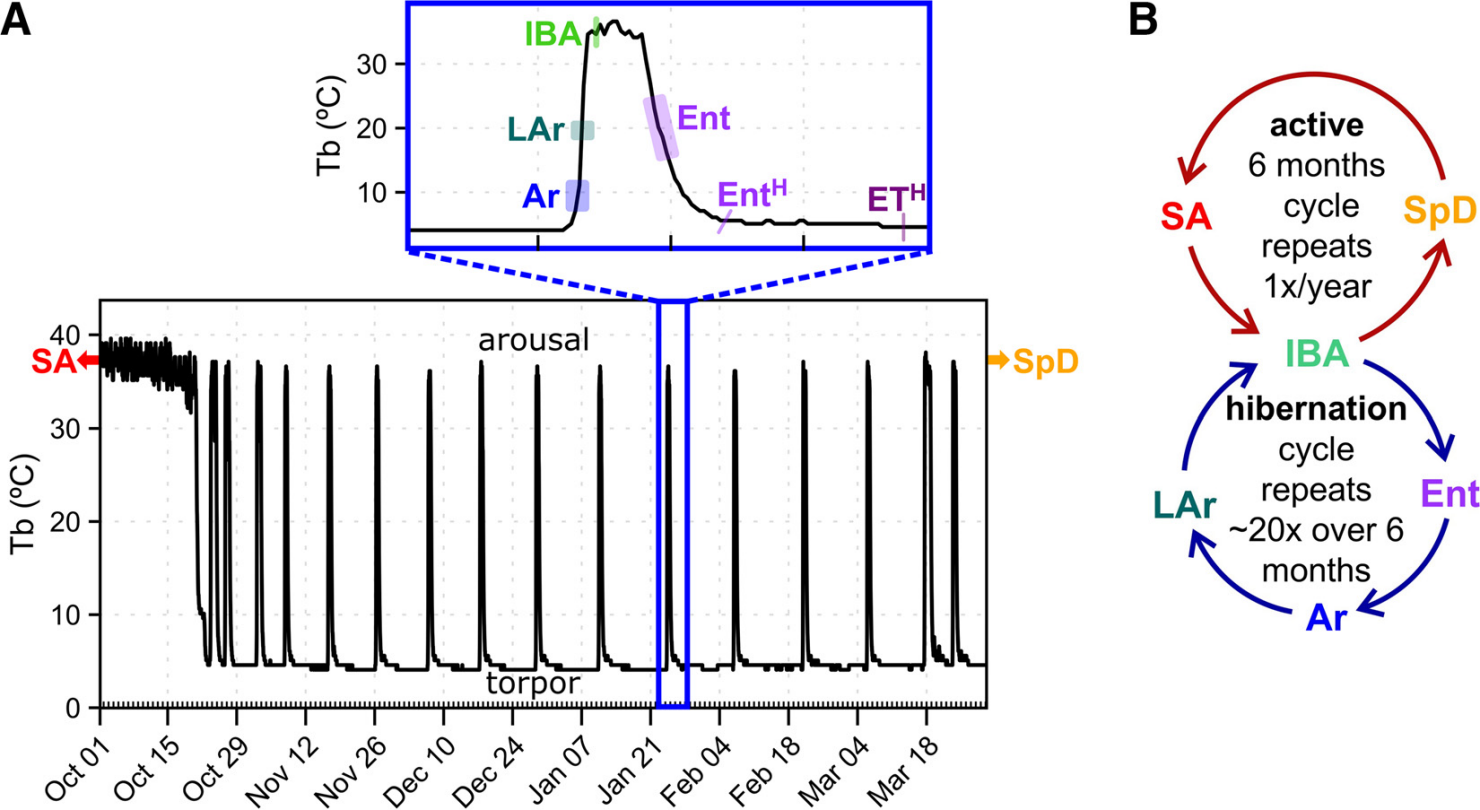
Hibernation rhythms with sample groups. *A*: plot of the body temperature (Tb) of a 13-lined ground squirrel (13-LGS) for 6 mo, from the beginning of October through the end of March, illustrates the heterothermy of hibernation; inner ticks mark days. Torpor, the multiday periods with Tb below 6°C and the short arousal periods that periodically restore Tb to near −37°C are indicated. The homeothermic groups, SA and SpD, precede or follow hibernation, respectively, as indicated by the labeled arrows; their Tb would look like the first week of October on this plot. The enlarged, 4 days inset of the indicated arousal from torpor (blue rectangle) defines the hibernation groups examined by RNA-Seq and histopathology, with highlighted regions showing the average ± SD of Tb (Ar, LAr, and IBA groups) or time (IBA group). Two samples used only for histopathology, one outlier Ent^H^ animal that had cooled below the typical Tb for Ent, and the unique early torpor animal (ET^H^) are indicated; additional animal details are provided in materials and methods and Supplemental Tables S1 and S2. *B*: depiction of two-cycle model of hibernation showing the single seasonal transition (red arrows) with embedded, numerous torpor-arousal cycles (blue). Ar, arousing; Ent, entrance; ET, early torpor; IBA, interbout aroused; LAr, later arousing; SA, summer active; SpD, spring dark.

Hibernation is a powerful model with unrealized potential to inform strategies for improving outcomes after AKI caused by ischemia and reperfusion injury and for kidney transplant with prolonged cold storage (8). In the 13-lined ground squirrels (13-LGS) studied here, multiday periods of torpor with ice-cold body temperature (Tb) are interrupted by ∼20 arousals over several months. Arousals are characterized by rapid rewarming to ∼37°C for less than 1 day before torpor reentry (Fig. 1). While in torpor, metabolic rate is 2%–5% of basal, heart rate declines from the euthermic 200–300 to 3–5 beats/min, and respiration decreases from 100–200 to 4–6 breaths/min (9). Animals arousing from torpor exhibit hyperactivation of metabolism, heart, and respiratory rates to rapidly rewarm (10). Although slowed metabolism during entrance into and throughout torpor appears to balance oxygen demand with delivery, the rapid rewarming that drives arousal from torpor begins with low oxygen tension (11) and very high metabolic demand (10)—conditions of IRI, and, in the kidney, AKI. The rewarmed hibernator maintains euthermic body conditions for several hours before initiating the next bout of torpor. In torpor, as in AKI, urine production effectively ceases, and serum creatinine levels increase, but the latter quickly normalizes in arousal (12), consistent with cyclical cessation and then return of renal function (13, 14).

AKI may resolve in adaptive repair with restored kidney function or progress to chronic injury and renal failure after maladaptive repair. We lack a comprehensive understanding of the key determinants of these dramatically different outcomes (1, 15–17). Recent progress at the cell and molecular level indicates that the metabolically highly active proximal tubule (PT) cells are particularly prone to damage by ischemic events or exposure to toxins and may thus be the primary sensors and effectors in both AKI and progression to CKD (18). Broadly, the early responses to a damaging insult include increased programmed cell death of proximal tubule cells, immune activation, increased DNA damage response, and cell proliferation, which, if maladaptive repair occurs, is followed by myeloid cell activation and pathological fibrosis (4, 15, 16).

Several recent studies have interrogated gene expression changes following injury using RNA-Seq (19, 20), single-nuclei, single-cell, or spatial transcriptomic techniques (17, 21), with a goal of identifying biomarkers and molecular determinants of the pathways to adaptive versus maladaptive repair. Of particular interest, immediate early (IE) response (IER) genes are activated on reperfusion in human (19), *Acomys* (22), and mouse kidneys (20) following injury, regardless of which end point (i.e., functional recovery or renal failure) is achieved. A later common pattern of dynamic gene expression is also associated with immune infiltration and activation, then fibrosis, in kidneys progressing to chronic injury and renal failure (19, 20, 23–25). Yet we still lack a complete understanding of the molecular events, specifically those occurring between the activation of IER genes and the immune-activation/fibrotic pathway, which confer healthy repair and recovery versus failed repair and disease progression.

We first examined 13-LGS kidneys for any evidence of accumulating damage after multiple rewarmings from torpor (IRI-like events). When no evidence of histopathology or immune response was found, we sought to define the protective mechanisms allowing 13-LGS to emerge each spring with a functionally intact renal system. For this, RNA-Seq was used to identify kidney gene expression changes among six groups that captured the most dramatic physiological transitions of hibernation: active animals pre- and posthibernation and hibernators entering, ending (beginning to re-warm), midrewarming, and fully rewarmed from torpor (Fig. 1). Both seasonal and torpor-arousal cycle-specific gene expression changes were found. Working in concert, these DE genes orchestrated adaptive repair of the kidney before the end of each of the many ∼12 h arousals that comprise a hibernation season. Thus, during normal hibernation, the 13-LGS failed to initiate the pathological activation of cell death, immune system, and fibrotic pathways, despite, as demonstrated by an outlier animal, being fully capable of inducing these well-known programs that lead to maladaptive repair and the progression from injury to chronic disease.

## MATERIALS AND METHODS

### Ground Squirrel Kidneys

13-LGS, *Ictidomys tridecemlineatus*, was obtained from the captive breeding colony at the University of Wisconsin, Oshkosh, WI, or from a commercial supplier, TLS Research (Bloomington, IL), as described (26). Assignment to physiological group was based on Tb monitored continuously by iButton and radiotelemetry (27, 28). All animals were anesthetized with isoflurane, exsanguinated, and then perfused through the heart with at least six blood volumes of ice-cold 0.1 M sodium phosphate, pH 7.2, before dissection. Kidneys were frozen in liquid nitrogen and then stored continuously at −80°C until RNA preparation. For histopathology, after clearing the blood, perfusion continued with 2–3 additional blood volumes of 10% formalin as described (28). Kidney pieces were embedded in paraffin after dissection and overnight fixation in 10% formalin at 4°C. Animal metadata are provided in Supplemental Tables S1 and S2. All animal work was approved by the University of Colorado Anschutz Medical Campus Animal Care and Use Committee.

### Histopathology

Five-micron sections were cut from the kidney paraffin blocks and stained in the University of Colorado Cancer Center Research Histology Core with periodic acid-Schiff (PAS) to score total kidney injury (29) and Picrosirius red (PSR) for fibrillar collagen to assess fibrosis (30). Images from PAS staining were collected at ×400 and features scored in Adobe Photoshop while viewing at 100%. The experiment began with *n* = 3 blinded samples in four groups: spring dark (SpD), interbout aroused (IBA), entrance (Ent) (one of these was an outlier, with low Tb, indicated by Ent^H^ in Fig. 1*A*), and a low Tb group with two Ar and one early torpor (ET) animal (Fig. 1, Supplemental Table S2); one of the SpD animals was later excluded because the tissue was inadequately perfused and fixed. For quantification of fibrillar collagen, PSR-stained sections from the same blocks were used to capture 1–3 high-quality ×40 mag (total) images, keying on the cortex and outer stripe of the outer medulla while keeping all camera settings identical. Images were captured on an Olympus BX51 microscope equipped with a 17 mp Olympus DP73 high-definition, color digital camera using CellSens software (Olympus, Waltham, MA). Images were imported into the next-generation Image J software, “SlideBook” (Intelligent Imaging Innovations, Denver, CO). Areas of importance were “segmented,” and then the percent of positive pixels/segmented area was determined by the software. Numbers reported for each animal are the average across all images for that animal (Supplemental Table S2).

### RNA-Seq

Kidneys from six stages representing key transitions of hibernation were used for RNA-Seq: two from the active, i.e., homeothermic, phase (Tb ∼37°C) of the year, summer active (SA, *n* = 6), fattening animals in August, and spring dark (SpD, *n* = 5), still housed in the hibernaculum after apparent emergence from hibernation (homeothermic 3–20 days since last torpor bout) and four from hibernation, i.e., the heterothermic phase of the year based on spontaneous changes in Tb: arousing (Ar, *n* = 5) from torpor with Tb 6.0°C–13.1°C; later arousing (LAr, *n* = 6), ∼45 min after Ar while still actively rewarming from torpor, Tb 16.2°C–21.7°C; interbout aroused (IBA, *n* = 5), within 2–3 h after Tb crossed 30°C during rewarming from torpor; entrance (Ent, *n* = 5) into torpor, Tb decreasing to 11.6°C–27°C after IBA; see also Fig. 1 and Supplemental Table S1. A 50–80 mg fragment of each frozen kidney was homogenized by polytron (Brinkmann) in 1.0 mL ice-cold TriReagent (Sigma). RNA recovered in the aqueous phase was further purified and Dnase treated on an RNAclean and concentrator column (Zymo). The eluted RNA was quantified by Nanodrop, and quality was assessed by Bioanalyzer; RIN values were 8.5 ± 0.7 (Supplemental Table S1). Initially, RNA-Seq data were collected from nine samples, three each from IBA, Ent, and SpD, as depicted in Supplemental Fig. S1, *experiment 1*. These purified RNAs were submitted to Genewiz, Inc. (Plainfield, NJ) for library preparation using the TruSeq Stranded mRNA Library Prep Kit (Illumina) and sequencing (151 nt paired-end reads, collected on an IlluminaHiSeq4000). The demultiplexed fastq files were returned to Colorado for analysis. A second set of 23 samples was added subsequently. These RNAs were converted to libraries (Universal Plus mRNA Sequencing Kit, Nugen) and sequenced (151 nt paired-end reads, collected on an Illumina NovaSEQ 6000) at the University of Colorado Genomics Core (*experiment 2*, Supplemental Fig. S1). The additional samples both increased the sample size for each physiological group and added three new groups (Ar, LAr, and SA). Both sample sets used poly(A) selection on oligodT beads and resulted in strand-specific sequencing libraries.

### RNA-Seq Data Analyses

Raw sequencing reads were trimmed with cutadapt to remove adaptor sequences, short sequences, and low-quality bases (-m17, -q10; 31). Trimmed reads were mapped to the 13-LGS mitochondrial genome with hisat2, quantified, and filtered (Supplemental Table S1; 32). The remaining reads were submitted to GEO (GSE227101) and mapped to transcript annotations on the HiC_Itri_2 genome (33) using Salmon (34) with -numBootstraps 50. These Salmon assignments were imported into R (35), summarized by gene using tximport (36), and filtered to retain only genes with rlog-normalized counts ≥7 in at least 4/5 individuals from at least one group. These values were used for random forest (37) analyses, which revealed a strong batch effect, such that samples from *experiment 1* were clearly separated from those sequenced in *experiment 2* (Supplemental Fig. S2*A*). We then used limma’s removeBatchEffect (38) to generate batch effect-corrected count tables. The adjusted count table was subjected to unsupervised random forest analysis, which confirmed the correction because the samples were no longer strongly clustered by batch (Supplemental Fig. S2*B*). These batch-corrected values were also used as input for variable selection before random forest with ntree = 100,000, ntreeIterat = 50,000,vars.drop.frac = 0.2, plotting, and WGCNA (39). Differentially expressed (DE) genes were defined as those with a likelihood ratio test (LRT) adjusted *P* value of < 0.001 across all states using DESeq2 (40) on the uncorrected count tables of 29 13-LGS after removal of outliers (*n* = 5 for all groups except SA, where *n* = 4). The model included terms to control for the effect of sex and for batch. DESeq2 was also used to calculate “shrunken” log2 fold changes (41) for DE genes across relevant pairwise transitions and normalized, transformed (rlog) count matrices (Supplemental Table S3).

### Cluster Analyses

For WGCNA, the batch-corrected pseudocounts were processed with ComBat (42) to remove effects of sex and then passed to WGCNA (v.1.68, 39) for module detection. Parameters were optimized to construct a signed network (TOMType = “signed,” networkType = “signed”), high sensitivity (deepSplit = 3), with more aggressive than default merging and reassignment (mergeCutHeight = 0.25, reassignThreshold = 1). Additional settings were minModuleSize = 30, minCoreKME = 0.5, minKMEtoStay = 0.4. Module-trait association quantification was used to identify modules that were significantly associated with the measured traits, and their correlation values were color-coded for plotting. For reference pattern clustering, we defined the 16 most common patterns informed by the WGCNA modules. Mean expression values were calculated for each gene in each state, and those values across the six states were tested for correlation with each of the reference patterns by calculating the Pearson correlation coefficient. Strongly correlated genes (*r* > 0.8) were assigned to their most highly correlated cluster. Genes with lower correlation values (*r* < 0.8) were assigned “no_cor.”

### Functional Annotation and Enrichment

Gene lists (clean_gene_symbol, Metascape, or updated_gene_symbol, DAVID, Supplemental Table S4) were annotated and analyzed for enrichment patterns using Metascape v3.5.20240101 (43) or DAVID Knowledgebase v2025_1 (44). For Metascape, default settings were used with *Homo sapiens* as background and clean_gene_symbol with and without the Multiple Gene Lists option. For DAVID, gene lists were submitted to functional annotation charts after selecting Gene_ontology (GO) options BP, CC, and MF (biological process, cellular component, and molecular function, respectively) _DIRECT. Annotations with *P* < 0.001 were retained. Specific functional information for individual genes was obtained from these annotations and the NCBI gene webserver unless a specific reference is provided.

### Deconvolution of RNA-Seq Data Using a Single-Cell RNA-Seq Reference

The bulk transcriptome from each 13-LGS was deconvoluted using a murine healthy kidney reference (45, GEO accession GSE107585) and a murine injured kidney reference (25, GEO accession GSE139107) using DISSECT (46). In brief, all confident 13-LGS homologs in mouse were identified by first retaining all HiC_Itri_2 transcript annotations (33) with valid human or murine gene symbols and then using a table of one-to-one human-mouse orthologs (47) with Ensembl v105 (48) to add murine gene symbols to 13-LGS genes with human gene symbols. Confident homologs with multiple genes in the 13-LGS transcriptome were summed to produce a count matrix with one entry per murine gene symbol. DISSECT was then used to deconvolute single-cell count matrices from GSE107585 and GSE139107. The annotated count matrix from GSE139107 was combined with PT cell annotations obtained from Dr. B. Humphreys (reflecting the cell labels shown in Fig. 2 of Ref. 25) and randomly downsampled to retain 25% of cells with each cell type/state label, with a minimum of 250 cells per label. DISSECT was run with default parameters except no gene/cell filtering, 1,000 cells per sample, and 16,000 total samples (GSE139107 only).

## RESULTS

### Histological Evaluation of Kidney Damage after Repeated Torpor-Arousal Cycles

Previous studies document that hibernator kidneys show little evidence of histopathological damage in torpor or after arousal (14). To our knowledge, however, the accrual of damage across the hibernation season (multiple cycles of “cold storage” and warm reperfusion) has not been examined. In captivity, 13-LGS undergoes ∼20 torpor-arousal cycles each fall to winter hibernation season yet emerge in spring with their renal systems functionally intact. Because AKI typically involves immune system activation and fibrosis regardless of the cause (15), we examined PAS- and PSR-stained kidney sections from 11 individuals, all of which had been hibernating for 3–7 mo and undergone 11–22 spontaneous arousals to Tb > 35°C from Tb < 6°C. We found no histological evidence of damage in the glomerulus and S1 and S2 proximal convoluted tubules or distal convoluted tubules and a lack of casts in the most susceptible S3 proximal tubules of the outer segment of the outer medulla (Supplemental Table S2). Moreover, there was no evidence of immune cell activation or infiltration, fibrosis, or other signs of renal damage regardless of the duration of hibernation or the overall number of arousal episodes. These results support and extend earlier findings of natural resistance to renal damage in hibernators and strengthen the conclusion that elucidating their protective mechanisms could reveal novel strategies to promote functional repair and prevent disease progression in both native and transplanted kidneys.

### RNA-Seq Data Analyses

To gain insight into mechanisms underlying renal protection in these hibernators, we collected RNA-Seq data from 32 13-LGS kidneys representing six key physiological transitions of hibernation (Fig. 1). After correction for a batch effect, three 13-LGS (K136, K137, and K113; Supplemental Fig. S2*B*) remained outliers compared with the others in their physiological groups, implying at least one other source of variation besides hibernation physiology among the samples.

### Cell Type Representation among Kidney Samples

The kidney is a complex structure with numerous distinct cell types (49, 50), raising the possibility that microanatomical differences, in addition to hibernation physiology, may account for some of the variation among samples. We assessed the representation of known renal cell types in our dataset by quantifying the average counts of mouse marker genes (48) across all samples. The marker genes for endothelial cells, podocytes, proximal tubules, distal convoluted tubules, collecting ducts, fibroblasts, novel 1 and novel 2 cells, and macrophages were all well-represented among our pass-filter genes. In contrast, seven of the eight immune cell marker genes expressed in mouse neutrophils, B and T lymphocytes, and natural killer cells were absent (Supplemental Fig. S3*A*). All four of these immune cell types were at least as abundant in mouse kidney as podocytes and both podocyte marker genes were detected in our RNA-Seq data, implying immune cells, except for macrophages, were underrepresented in 13-LGS kidneys compared with mouse.

**Figure 2.**
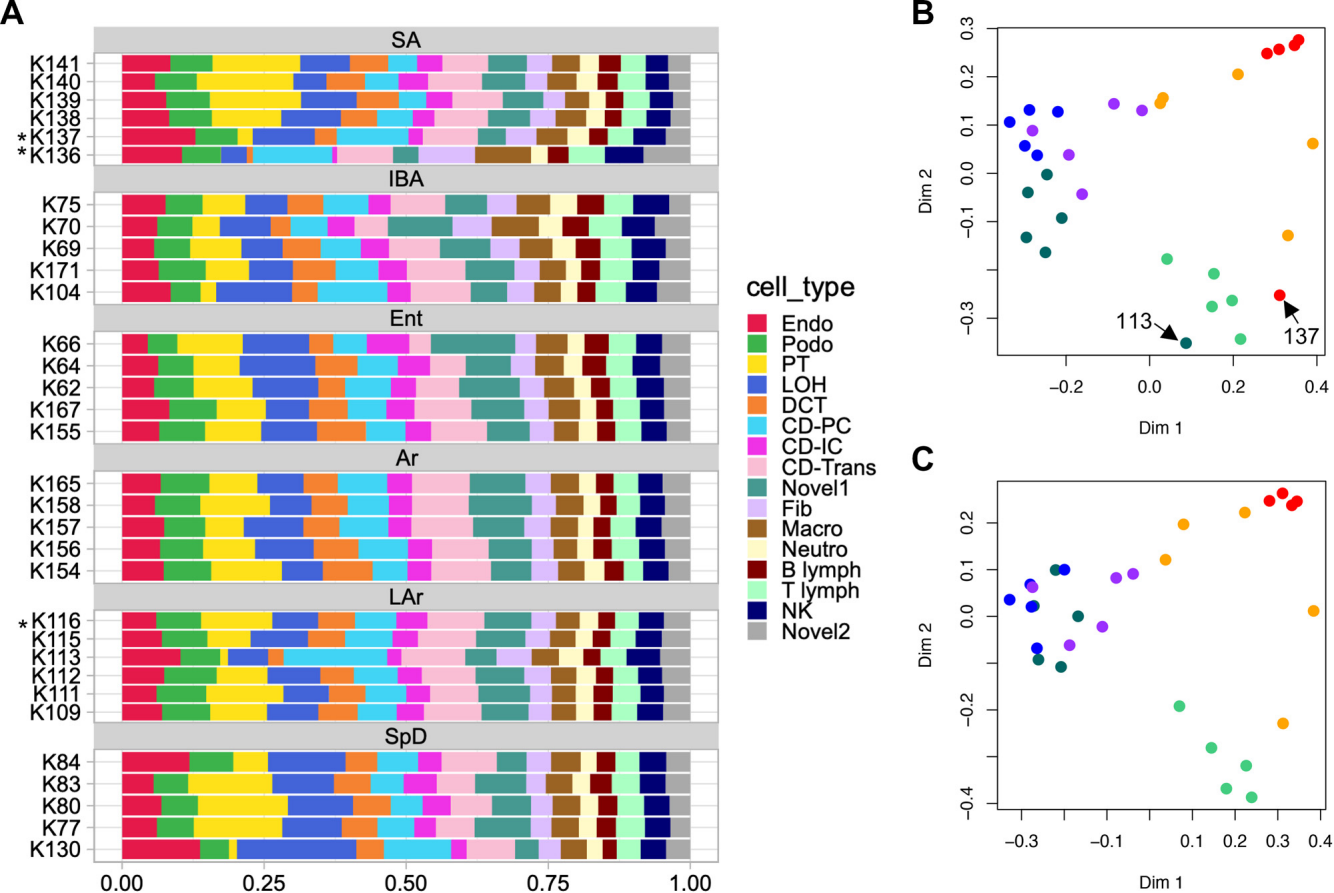
Variation among 13-LGS kidney samples. *A*: estimated cell type proportions in individual kidneys, grouped by hibernation state, based on DISSECT (45) deconvolution using mouse scRNA-Seq data (45); *the three outliers. Cell types are as defined in Ref. 45. Endo, endothelial, vascular, and descending loop of Henle; Podo, podocyte; PT, proximal tubule; LOH, ascending loop of Henle; DCT, distal convoluted tubule; CD-PC, collecting duct principal cell; CD-IC, collecting duct intercalated cell; CD-Trans, collecting duct transitional cell; Novel1, novel cell 1; Fib, fibroblast; Macro, macrophage; Neutro, neutrophil; lymph, lymphocyte; NK, natural killer cell; Novel2; novel cell 2. *B* and *C*: two-dimensional scaling plots of 13-LGS samples generated by supervised random forest clustering of all pass-filter kidney genes: (*B*) excluding SA-K136, labels highlight the two remaining outliers, LAr-K113 and SA-K137 and (*C*) excluding all three outliers: SA-K136, LAr-K113, and SA-K137. 13-LGS, 13-lined ground squirrel; LAr, later arousing; SA, summer active.

Independently, the relative proportions of renal cell types for individual 13-LGS were estimated from their kidney RNA-Seq data using DISSECT(45) trained on mouse single-cell RNA sequencing (scRNA-Seq) data (48). Cell type variation was apparent to a greater (SA and SpD) or lesser (Ent and Ar) extent within each hibernation group (Fig. 2*A*). One of the abovementioned outliers, K136, stands out with almost no detectable proximal tubule cells and substantially increased immune cell types and fibroblasts.

### Evidence for Immune Activation and Fibrosis in Outlier K136

Significantly, all three of the immune cell marker genes that were detected in all 13-LGS kidneys, *C1qa* and *C1qb* from macrophages and *Cd79a* from B lymphocytes, were substantially elevated compared with the mean of the other SA animals in the outlier SA sample, K136 (Supplemental Fig. S3*B*). To determine whether the missing immune cell marker genes were filtered from our data set due to low coverage or simply not annotated in this species, we examined them using the genome browser (33). All seven of the missing immune cell genes were annotated in 13-LGS. Moreover, each demonstrated substantially elevated coverage in K136 (Supplemental Fig. S3*C*). To obtain a more global view of the altered gene expression pattern in this animal, we identified functional enrichments in the 100 most over- and underrepresented genes in K136 relative to the mean values of the other SA animals. These enrichments reflected the well-described characteristics of maladaptive repair and kidney disease in other species (4, 51), specifically, infiltration and activation of immune cells and onset of fibrosis at the expense of normal kidney functions of transport and metabolism (Supplemental Fig. S3*D*). After searching husbandry records that were not consulted when selecting samples for RNA-Seq, we found only K136 was noted to have gross anatomical aberrancies at dissection; specifically, a shrunken left kidney with an apparent clot on or connected to it and an enlarged right kidney. We therefore excluded K136 from further analyses of the normal kidney’s response to hibernation.

### Two Remaining Outliers

When the kidney samples were clustered by random forests after removing K136, two outliers, K113 and K137, remained (Fig. 2*B*). These two samples were also extreme examples of apparent cell type variation within their groups (Fig. 2*A*), although enrichment analysis of genes over- and underrepresented from the means of the other LAr (K113) and SA (K137) animals did not indicate immune activation or other features of the progression from AKI to CKD as found for K136. Instead, these two appear to be timing variants, i.e., further along in their state transition (Supplemental Fig. S4). Excluding these two individuals further improved clustering of the remaining samples by hibernation physiology (Fig. 2*C*).

### Quantitative Changes in Kidney Gene Expression across Physiological Transitions

Differentially expressed (DE) genes in hibernation were quantified in the remaining 29 samples representing six physiological states using DESeq2. We also considered sequential pairwise changes across the seasonal and torpor-arousal cycle transitions of hibernation (Table 1, Supplemental Table S3). These analyses revealed 3,446 DE genes (*q* < 0.001), comprising 26.8% of the 12,847 total pass-filter genes examined. Consistent with the random forest clusters, the greatest number of pairwise DE genes, 882, occurred as animals transitioned from the active season into the hibernation season (SA to IBA), followed by 686 changing across the brief interval between LAr and IBA (∼3 h), as animals recovered active gene expression (52–54) while rewarming from torpor. During torpor-arousal cycles, transcripts for more genes increased in IBA than in either LAr or Ent, indicating a burst of active gene expression at this early timepoint after Tb recovers from a torpor bout, as well as a dynamic gene expression program that unfurls across the brief (∼12 h) warm interval of LAr through IBA to Ent. In contrast, when IBA was compared with either SpD or SA, more genes were active during the active season (higher in SA or SpD than IBA); this finding is consistent with a generally depressed renal function and conservation of energy throughout the hibernation season.

**Table 1.** Summary of kidney DE genes across relevant pairs of physiological transitions

	SA/SpD	IBA/SA	Ent/IBA	Ar/Ent	LAr/Ar	IBA/LAr	SpD/IBA
Total DE	184	882	411	31	54	686	492
# increased	102	328	159	15	54	459	300
# decreased	82	554	252	16	0	227	192
# ≥ 2x increased	24	36	10	14	34	128	119
# ≥ 2x decreased	13	261	8	8	0	50	33
fraction DE ≥ 2x	0.20	0.34	0.04	0.71	0.63	0.26	0.31
Max FC increased	16.0	14.4	4.0	36.6	22.5	54.2	7.8
Max FC decreased	2.4	83.1	2.5	27.6	NA	46.0	13.3
Gene ID unknown	28	102	44	18	6	98	54
Fraction unknown	0.15	0.12	0.11	0.58	0.11	0.14	0.11

Counts of significant (*q* < 0.001) DE genes from DEseq2 analysis of 13-LGS kidney RNA-Seq. Physiological states are as defined in materials and methods and Fig. 1 (relevant pairs as depicted in Fig.1B), full DEseq2 results are in Supplemental Table S3; unknown, updated_gene_symbol = NA. 13-LGS, 13-lined ground squirrel; Ar, arousing; DE, differentially expressed; Ent, entrance; IBA, interbout aroused; LAr, later arousing; SA, summer active; SpD, spring dark.

Several other noteworthy observations emerged from this quantitative analysis of gene expression changes among pairwise transitions of hibernation. There were relatively few changes across the torpor bout (Ent to Ar) despite this being by far the longest phase in the torpor-arousal cycle (≥ 5 days; Fig. 1*A*). This interval spanning the torpor bout also had the highest proportion of genes that both differed by more than twofold and were unknown (Table 1). In sharp contrast, the short interval from Ar to LAr (<1 h), which again had a relatively small number of DE genes, had the highest proportion (100%) of genes increased. A relatively large proportion of the increased genes did so by at least twofold, but in sharp contrast to the torpor interval, these were largely known, protein-coding genes (Supplemental Table S3). Consistent with the random forests clustering, this quantitative analysis of gene expression indicates strong seasonal and torpor-arousal cycle effects on gene expression.

### Gene Coexpression Clusters and Their Functional Enrichments

**Figure 3.**
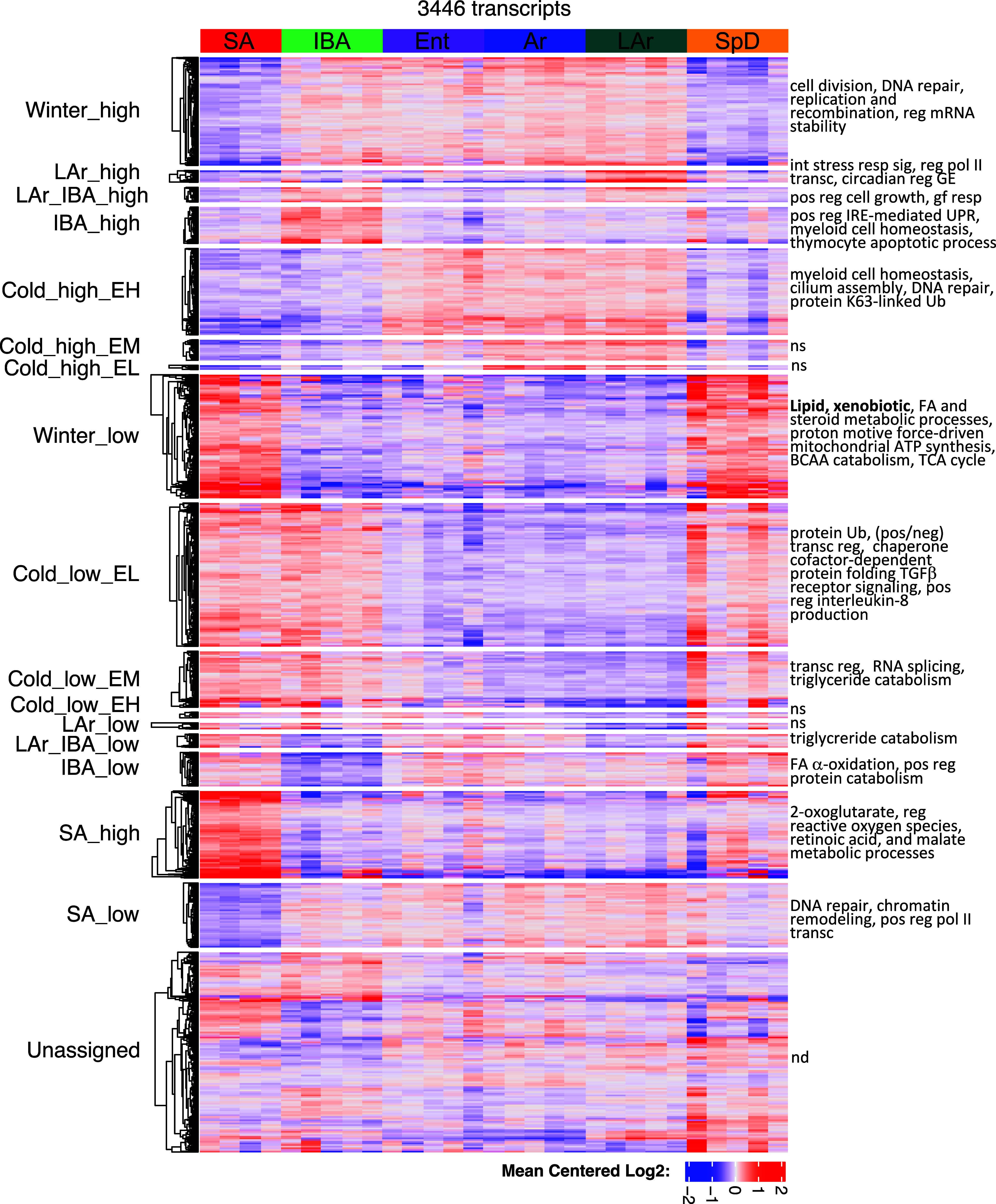
Heatmap of kidney DE genes grouped by coexpression dynamics and physiological state. Relative gene expression values for each gene in each kidney were arranged vertically by coexpression cluster on *left*, horizontally by hibernation state. Top enriched (*P* < 0.01) GO Biological Process terms from DAVID webserver (44) are indicated at *right*, in bold if Benjamini < 0.001, ns, no significant enrichments; nd, not done. See Supplemental Table S4 for additional enrichment annotations. The number of genes in each cluster were Winter_high, 366; LAr_high, 41; LAr_IBA_high, 51; IBA_high, 124; Cold_high_EH, 293; Cold_high_EM, 70; Cold_high_EL, 16; Winter_low, 418; Cold_low_EL, 485; Cold_low_EM, 190; Cold_low_EH, 20; LAr_low, 22; LAr_IBA_low, 46; IBA_low, 115; SA_high, 295; SA_low, 218; no_cor, 676. BCAA, branched-chain amino acids; DS, double-strand; FA, fatty acid; GE, gene expression; GO, Gene Ontology; gf, growth factor; int, integrated; IRE-med, IRE (inositol-requiring enzyme 1)-mediated; pol, polymerase; pos, positive; reg, regulation; resp, response; sig, signaling; TCA, tricarboxylic acid; transc, transcription; UPR, unfolded-protein response.

Phenotypic parameters (Supplemental Table S1) were correlated to gene coexpression clusters using WGCNA (39). As observed earlier, Tb, heterothermy, and some of the distinct or paired hibernation states, particularly LAr, were most strongly correlated with transcriptome dynamics (Supplemental Fig. S5). Based on these observations, we defined 16 coexpression patterns and clustered the DE genes based on their correlation with these patterns, capturing 80.4% of the kidney DE genes. A heatmap of the relative abundance of genes within each cluster again highlighted the strong effects of season and torpor-arousal cycle on gene expression dynamics (Fig. 3). The majority (65%) of the DE transcripts fell into one of two patterns: *1*) low in both active states (SA and SpD) but elevated in one to all four hibernation states (IBA, Ent, Ar, and LAr) or *2*) elevated in both active states but low in one to all four hibernation states. The remainder of the classified genes, in SA_high or SA_low, appeared to be best explained as a special case of the seasonal categories Winter_low and Winter_high, respectively, where SpD individuals had variably recovered active-season levels of these transcripts after emerging from hibernation. The results of gene enrichment analyses (GO Biological Process) further supported this view: like the Winter_low cluster, genes classified as SA_high were most enriched for metabolic pathways. In contrast, like the Winter_high cluster, genes classified as SA_low were enriched in terms DNA repair and those affecting gene expression including mRNA expression and stability and chromatin remodeling (Fig. 3, Supplemental Table S4).

Several features emerged from gene enrichment analyses of these coexpression clusters. The 784 seasonally DE genes, i.e., those increased or decreased in both active states compared with the four hibernation states, comprised 28.3% of the genes with defined coexpression patterns. Genes elevated throughout hibernation (Winter_high) were enriched for functions related to cell division and DNA replication, DNA repair and recombination, and the regulation of RNA stability. In contrast, the functional enrichment of genes seasonally decreased in hibernation (Winter_low) was dominated by metabolic processes. When the SA_high and SA_low genes were merged with Winter_low and Winter_high, respectively, based on their similar functional enrichments (noted earlier), the proportion of DE genes that was seasonally altered increased to nearly 47%. The remaining DE genes cycled within the torpor-arousal cycle, either uniquely within hibernation (21.5%) or increased to the level found in active animals during one or more of the hibernation states (31.7%). Because transcription and mRNA processing effectively cease at the low Tb of torpor (52–54), rewarming appeared to provide a synchronous signal that activates a serial gene expression program that unfolds over the ∼12 h warm period, beginning in LAr, followed by IBA and ending in Ent.

Among the 595 dynamic kidney genes (21.5%) that were uniquely activated in hibernation, the first-on LAr_high genes were enriched for functions in the integrated stress response pathway, regulation of mRNA transcription and circadian regulation. Next, the LAr_IBA_high cluster was enriched for genes involved in the positive regulation of cell growth and response to growth factors. Then in IBA_high, the increased genes were involved in the positive regulation of the unfolded protein response (UPR), myeloid cell homeostasis, and thymocyte apoptosis. Finally, as animals were beginning to reenter torpor, the increased genes in Ent were functionally enriched in cilium assembly, DNA repair, myeloid cell homeostasis, and a form of ubiquitin modification (K63-linked) associated with membrane protein trafficking and quality control in the endolysosomal system rather than protein turnover (55).

Slightly over half of the 878 genes that were restored to active levels at some point during arousal exhibited the Cold_low_EL pattern. As with genes in the IBA_high cluster, these genes were enriched in pathways related to gene expression (transcriptional regulation), proteostasis (protein ubiquitination and protein folding), and immune system function (TGFB-receptor signaling and regulation of IL-8 production), all strongly decreased before torpor reentry. Genes with roles in transcriptional regulation, RNA splicing, and triglyceride catabolism increased in IBA but remained elevated longer (Cold_low_EM). The last group of genes, those increased at the end of the warm period just before torpor reentry found in clusters LAr_IBA_low and IBA_low, were enriched for functions in triglyceride catabolism, fatty acid oxidation, and positive regulation of protein catabolism.

### Comparison with Mouse IRI

Several studies report gene expression changes associated with AKI and the progression to CKD (17, 21). We found two of these to be most relevant to our hibernation data because they captured more than one time point within the first 24 h (20, 25).

**Figure 4.**
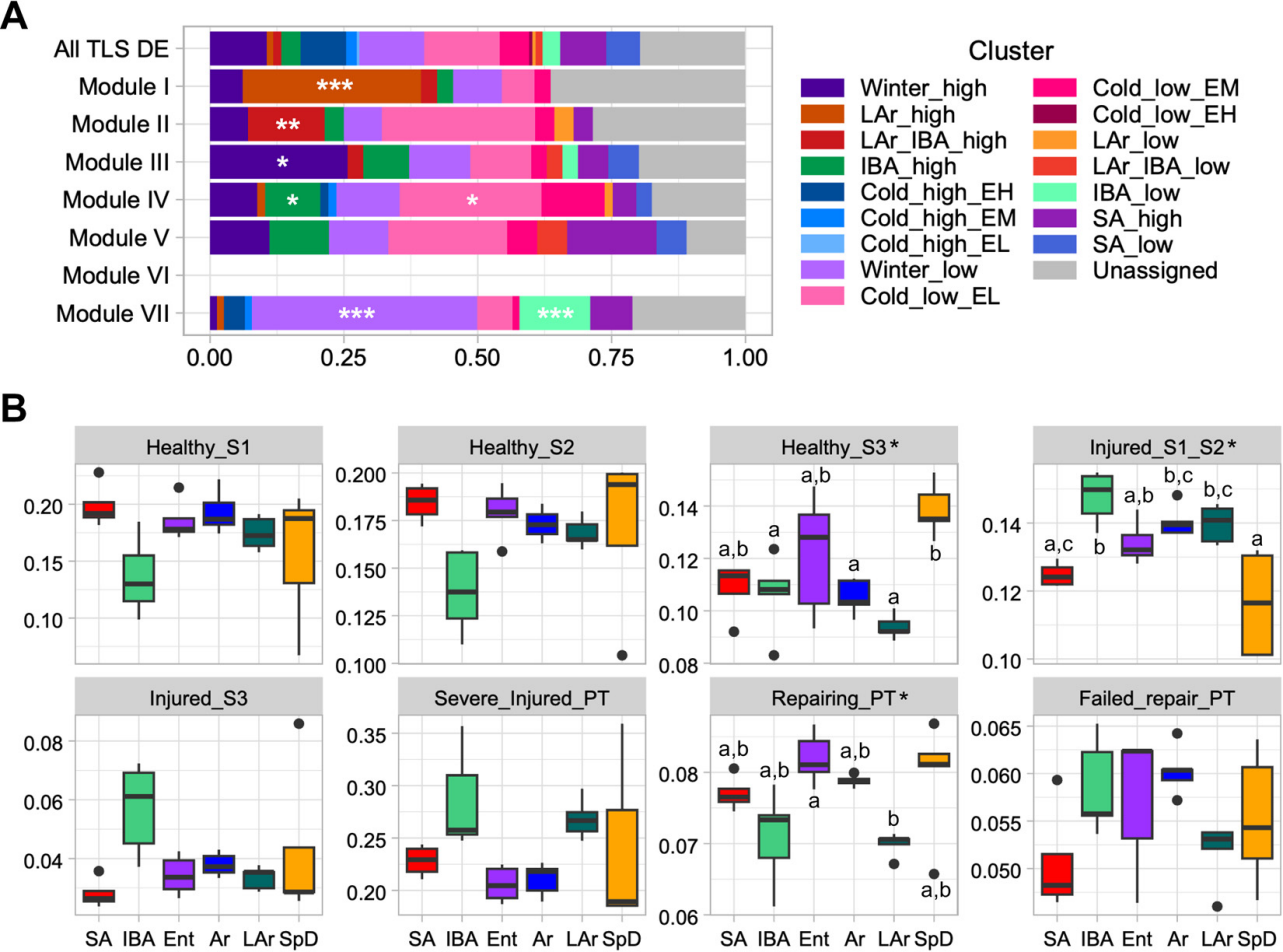
Comparison of DE gene expression in hibernation and after ischemia-reperfusion (IR) injury in mouse kidney. *A*: bars show the proportion of all 13-LGS DE genes in each coexpression cluster and those overlapping with genes in each post-IR injury module in the mouse. Asterisks mark clusters with overrepresentation based on Fisher’s exact test with *P <0.05, **P <0.01, and ***P <0.001. *B*: DISSECT reconstructions plotted as proportions of healthy, injured, repairing, and failed-repair PT cells (25) by hibernation state. Dots indicate outliers, asterisks indicate ANOVA *P* < 0.01, small letters near boxes identify states with different cell proportions (Tukey’s HSD, *P* < 0.01). See Table 2 for additional details. 13-LGS, 13-lined ground squirrel; DE, differentially expressed; HSD, honestly significant difference.

The response of the mouse kidney to IRI was quantified by RNA-Seq of samples taken 2 h to 12 mo postreperfusion; this study defined seven coexpression modules with distinct time trajectories (20). We quantified 13-LGS DE genes shared with these modules and then asked whether any hibernation coexpression clusters were enriched (Fig. 4*A*, Table 2, Supplemental Table S4). *Module 1* genes are rapidly and transiently expressed from 2 to 4 h after IRI and strongly overlapped with 13-LGS LAr_high genes, those first expressed as the animals emerged from torpor. All three gene sets (mouse IRI *module I*, 13-LGS LAr_high, and the genes common to both) were dominated by terms related to transcription and stress response and reflected activation of a robust immediate early response (Fig. 4*A*, Table 2, Supplemental Table S4). *Module II*, with genes related to cell death and response to wounding in mice, was most enriched in LAr_IBA_high genes, consistent with their early activation in both IRI and arousal from torpor. However, in mice, *module II* genes remained elevated for weeks after reperfusion, whereas the LAr_IBA_high genes during hibernation returned to baseline before Ent, less than 12 h later. The top two most enriched GO:BP terms in their common gene set were positive regulation of cell population proliferation and negative regulation of apoptotic process. Winter_high genes were overrepresented in *module III*, consistent with the enrichment of cell cycle, cell division, and mitosis genes in both, although *module III* genes took 2–3 days to increase following IRI in mice and then returned to baseline after 1 wk, in contrast to their months of elevated expression throughout hibernation. *Module IV* genes were most overrepresented in the 13-LGS IBA_high group, but Cold_low_EL genes were also enriched (*P* = 0.03). The functional enrichment terms associated with the 13-LGS genes in *module IV* included cell adhesion and response to wounding, but no terms related to inflammation, as found in the mouse IRI genes. Here again, the time course differed—*module IV* genes increased only after 1 day then remained elevated for more than 1 wk, whereas Cold_low_EL genes and IBA_high genes increased 4–5 h after Tb began to increase during arousal and were already decreased within 12 h as animals reentered torpor. *Module V* in the mouse is dominated by immune response genes, but these increase only after 1 wk and remain high for months. There are no significant overlapping patterns among the 13-LGS DE genes, but the common genes in *module V* were also enriched for immune system function (Supplemental Table S4). *Module VII* genes in the mouse IRI model decrease rapidly (2–4 h) and then recover within 24 h after reperfusion. Interestingly, the 13-LGS coexpression clusters most enriched with *module VII* genes were those in Winter_low and IBA_high, their shared functional enrichments with *module VII* genes included mitochondria, oxidoreductase, symporter and transporter activities, and fatty acid oxidation. Taken together, the results of this comparison indicate that arousal from torpor in 13-LGS induces a gene expression pattern in kidney that begins with an IE response very similar to that of a mouse following IRI but then strongly deviates temporally, although not functionally.

**Table 2.** Mouse IRI injury module genes in 13-LGS hibernation coexpression clusters

Mouse IRI Module (Gene No.)	Time DE (Mouse)	Mouse IRI Module Functional Enrichments	Most Enriched Hibernation Cluster	*P* value (Fisher’s exact Test)
I (33/101)	↑2 and 4 h, ↓ by 24 h	Transcription, IE response genes	LAr_high	1.24E-11
II (28/99)	↑2 and 4 h, remain ↑ >1 wk	Death, apoptosis, response to wounding	LAr_IBA_high	1.37E-03
III (34/371)	↑2–3 days, ↓ by 7 days	Cell cycle, cell division, mitosis	Winter_high	0.032
IV (68/516)	↑1 day, remain ↑ >1 wk	Cell adhesion, inflammatory response	IBA_high	0.017
V (18/352)	↑7 days, remain ↑ months	Immune response, leukocyte activation	IBA_high	0.154
VI (0/82)	↑6 and 12 mo	Immune-related	No overlap	na
VII (76/405)	↓2 and 4 h, ↑ by 24 h	Oxidation-reduction, AA catabolism	Winter_low	1.33E-07

Mouse data from Liu et al. (20), see also Fig. 4. Numbers in the first column are number of 13-LGS DE in module/number in module. 13-LGS, 13-lined ground squirrel; DE, differentially expressed; IRI, ischemia-reperfusion injury.

**Figure 5.**
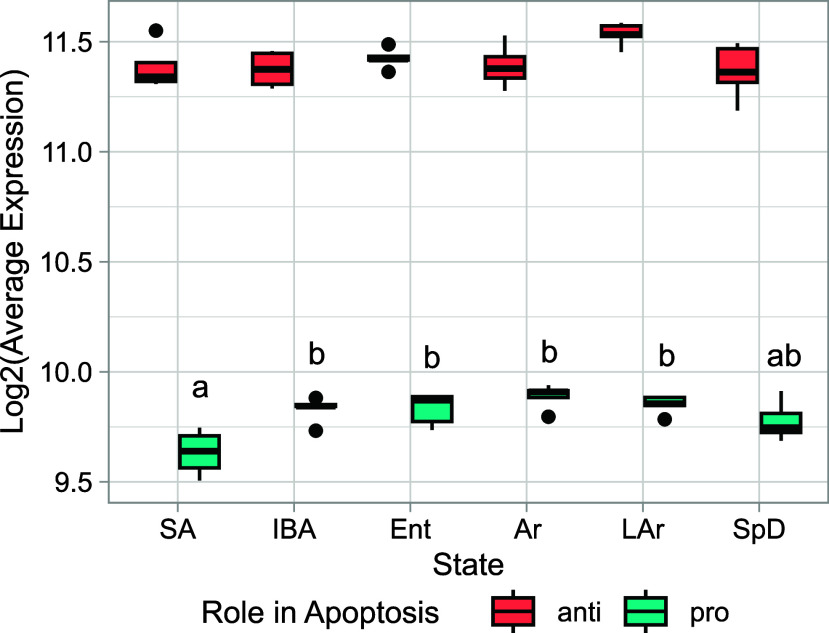
Elevated expression of antiapoptotic genes compared with proapoptotic genes in 13-LGS kidney. Boxplots of average relative abundance of key antiapoptotic (peach, *top*) and proapoptotic (blue, *bottom*) genes, plotted individually in Supplemental Fig. S6. Average count data from the 29 samples remaining after outliers K136, K137, and K113 were removed, only the proapoptotic genes were DE across hibernation states, ANOVA *P* < 0.001, letters indicate groups that differ (Tukey’s HSD *P* < 0.01). 13-LGS, 13-lined ground squirrel; DE, differentially expressed; HSD, honestly significant difference.

More recently, kidney responses to IRI captured by single-cell or nuclei RNA-Seq in mice reveal a range of cell states spanning healthy, repairing, injured, and failed repair cells of the proximal tubule (25). These characteristics were also identified in other datasets after deconvoluting bulk sequence data from human, mouse, and rat (25). When DISSECT was used for deconvolution of our 13-LGS RNA-seq data, injured PT cells were highest in IBA, whereas repairing and healthy PT cells were higher in Ent (Fig. 5*B*). These kinetics are consistent with torpor and rewarming from torpor, causing sufficient damage to initiate a modest, transient injury response immediately upon rewarming, which leads to rapid repair and restoration of structure and function of the kidney over the short 12 h warm period before the animals enter another bout of torpor.

### Relative Abundance of Antiapoptotic and Proapoptotic Genes

The onset of gene expression in LAr is widely characteristic of IRI damage response in kidneys from a variety of species, including human (25), yet in these hibernators, the serial gene expression program that unfolds afterward leads quickly to functional repair and avoids the widespread cell death, inflammation, and fibrosis caused by maladaptive repair. Cell death is an important component of the kidney’s response to IRI injury and occurs in both adaptive and maladaptive repair pathways (56). Earlier work indicates that neither apoptosis nor necrosis is elevated in 13-LGS kidney in IBA animals compared with those in SA or torpor (13).

Annotations related to cell death pathways were not highly enriched among the DE genes in our data set (Fig. 3, Supplemental Table S4); only one term in the coexpression clusters, thymocyte apoptosis, appeared in the IBA_high group. When seasonal effects were discounted, i.e., in the pairwise comparisons, a few more terms emerged, again mainly in the genes increased in IBA (upIBAvLAr) and strictly confined to the mechanism of apoptosis. There was also some evidence of increased death-related pathways in the overlap with the mouse data (LAr_IBA_high genes in mouse *module II*; Fig. 4 and Table 2), but the number of genes was small. Unlike the mouse, where the death genes remain elevated for at least 1 wk, these decreased within 12 h, before Ent, suggesting that the 13-LGS have a mechanism that biases cells toward survival or checks death pathway activation in IBA. Although we were unable to identify a specific gene to propose for such a role, we found elevated expression of several antiapoptotic genes compared with proapoptotic genes in the dataset (Fig. 5, Supplemental Fig. S6).

### Dynamics of Gene Expression across Arousal from Torpor Is Reminiscent of the 12-h Circatidal Rhythm

**Figure 6.**
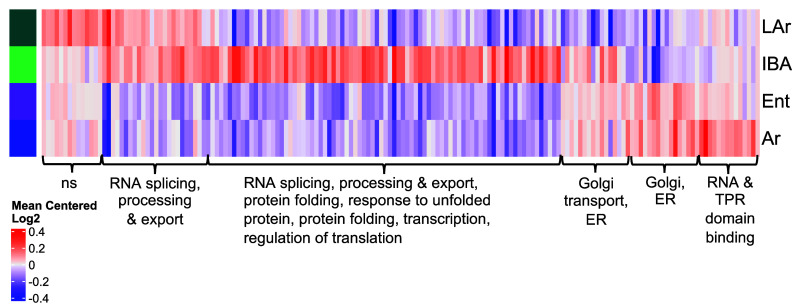
Kidney expression of 12-h genes across the torpor-arousal cycle in hibernating 13-LGS. Heatmap plots the relative abundance of 12-h genes identified in mouse liver (57) in hibernating kidney samples from the torpor-arousal cycle. Top enriched Gene Ontology (GO) terms found in each temporally distinct group of coexpressed genes are indicated. Only one gene has been reported to exhibit a 12-h rhythm in the kidney, *Hspa1b* (58). This gene is DE in our dataset and belongs to the Cold_low_EL coexpression cluster. 13-LGS, 13-lined ground squirrel; DE, differentially expressed; ER, endoplasmic reticulum; TPR, tetratricopeptide repeat.

Most functional enrichment annotations among the genes that varied across the torpor-arousal cycle related to universal cell physiology, i.e., synthesis of transcripts and proteins (Supplemental Table S4). Moreover, the genes for these crucial cell functions appeared to be temporally ordered across the hibernator’s brief warm arousal (Fig. 3). Functional enrichments related to gene expression, specifically transcription, RNA splicing, processing, and transport, as well as protein folding and processing through the endoplasmic reticulum and the Golgi, similarly characterize the biological rhythm known as the circatidal or 12-h rhythm. The majority of gene products are expressed relatively early in the 12-h rhythm, but there is a progression over the 12 h window that mirrors the flow of genetic information through the steps described by the central dogma (57). To examine our dataset for known 12-h genes, we selected all 12-h genes defined in mouse liver (57) that appeared in our dataset with LRT_padj < 0.05. These were plotted by temporal expression pattern (Fig. 6), and then the genes in each coexpression cohort were tested for functional enrichment. As with the 12-h rhythm, the first-on genes (increased in LAr and IBA) were enriched for terms related to RNA biogenesis, whereas the protein trafficking-related genes increased later (IBA and Ent, or just Ent). Moreover, just one gene is documented to cycle with 12-h rhythmicity in the kidney (58). This gene, *Hspa1b*, was Cold_low_EL; its elevated expression during IBA in the torpor-arousal cycle is analogous to its expression early in the 12-h rhythm and concordant with its role in protein folding.

## DISCUSSION

Torpid 13-LGS have elevated serum creatinine and decreased urinary output, thereby exhibiting the defining characteristics of AKI (13, 14). But, like torpor itself, this apparent AKI is transient, reversing periodically when the animals rewarm. Here, we show that the hibernator fails to develop the histological or molecular characteristics of AKI and progression to CKD even after numerous torpor-arousal cycles that alternate multiday periods of low blood flow at 4°C with rapid reperfusion and rewarming to 37°C. The hibernating 13-LGS thus avoids, both episodically during each arousal (13, 14) and seasonally after a score of them (Supplemental Table S2), the classic pathophysiological signs of kidney damage and disease in the transition from AKI to CKD, namely widespread cell death followed by immune infiltration, activation, and fibrosis (17, 59, 60).

To uncover the molecular mechanisms conferring the hibernator’s resistance to AKI and progression to CKD, we identified DE kidney genes across six physiological states of the annual cycle between activity and hibernation with its embedded torpor-arousal cycle. Consistent with the observed restoration of normal renal function and lack of histopathology after each torpor bout during IBA, as well as the end of hibernation, many genes associated with kidney damage and progression to disease in other organisms were unchanged or filtered due to low expression, including *Lcn2* (*Ngal*), *Vcam1*, *Dcdc2a*, *Sema5a* (17), and *S100a8/9* (61). One 13-LGS RNA-Seq library, however, provided an exception to this result. In the SA outlier, K136, numerous genes associated with immune activation and fibrosis increased, whereas genes encoding kidney transport and metabolic functions decreased (Supplemental Fig. S3). These changes are found weeks to months after kidney injury and are characteristic of failed repair in mouse models and in human transplant failure and other forms of CKD (19, 20, 25, 62). The contrast between this animal and the others demonstrates that the hibernating 13-LGS’s resilience is not due to a lack of the relevant biology, but to a highly effective, context-dependent mechanism that accelerates adaptive repair such that it is completed during each arousal, in ∼12 h.

After removing this CKD-like sample and two other outliers, which appeared to be timing variants (Supplemental Fig. S4), 3,446 hibernation-related DE genes were identified. The DE genes clustered into multiple coexpression patterns with clear effects of both season and torpor-arousal cycle on the kidney transcriptome (Figs. 2, 3). These results support and extend findings of gene expression in the brain (33), liver (52), and adrenal gland (63), all of which demonstrate the value of multiple, precisely timed samples for studies of molecular dynamics in hibernation. It is noteworthy that transcription (53) and processing (52) of mRNAs effectively cease at the low Tb of torpor, as do protein synthesis (64, 65) and degradation (66). Consequently, few regulated gene expression changes occur across the torpor bout, although it is by far the longest time interval in the torpor-arousal cycle (Figs. 1 and 3, Table 1).

Keeping the effects of low Tb on gene expression in mind while considering these kidney RNA-Seq results, it is not surprising that the smallest number of DE genes were found in the sampling interval that spans the torpor bout, Ent to Ar (Table 1, Supplemental Table S3). As expected, the few protein-coding mRNAs that were DE in this interval decreased and were thus less stable than the bulk polyadenylated RNA pool. The RNAs increased across this interval were all unknown except *Rpph1* (the RNA subunit of RNase P). Like *Rpph1*, they appear to be short ncRNAs that are processed through the nuclear exosome; inactivity of this RNA degradation pathway during torpor allows these otherwise transiently polyadenylated forms to accumulate (54). In contrast, only increased DE genes were found across the next sampling interval of the torpor-arousal cycle, which is also the shortest (45 min–1 h), Ar to LAr; these mostly known, protein-coding genes that all increased in LAr likely reflect that the kidney had finally rewarmed sufficiently for mRNA transcription (53) and processing (52) to resume. Here we saw rapid, transient, and relatively large-fold inductions of genes dominated by IER genes and other transcription factors (Fig. 3, Supplemental Tables S3 and S4). Rapid induction of IER genes following reperfusion (Fig. 4*A*) is widely shared among kidney (and other organ) injury models (19, 20, 22, 25, 67). The next interval, from LAr to IBA, is also relatively short, 1.5–3 h, during which time additional transcription factors increased, along with genes related to proteostasis, cell division, DNA repair, apoptosis, and immune activation. It is noteworthy that these changes in IBA are of lower magnitude, likely reflecting a divergence in response among the kidney’s dozens of cell types compared with a more synchronous response earlier in the interval of active gene expression. This trend toward lower fold change continues to the end of the warm period, i.e., across the IBA to Ent interval, where the number of DE genes also decreased. As Tb again fell to temperatures that no longer supported production of mature mRNAs, the elevated Ent transcripts were enriched in components of cilia and mitochondria, key structures for kidney function.

Current understanding of gene expression changes following IRI in the kidney is based on experiments in mice (20, 23–25, 61, 68–72) or data collected from biopsies of human kidney, largely from transplant patients (19, 51). Only a few mouse datasets have multiple samples within the first 24 h, and the human transplant samples are even more limited, with no more than one sample within the window most relevant for comparison to the 13-LGS samples. In all IRI cases studied, activation of a stress response beginning with increased IER genes is the earliest event after reperfusion, followed by genes involved in cell death and repair. For adaptive repair, injured cells may escape death and repair themselves, or nearby cells may dedifferentiate, enter the cell cycle, replicate, and then redifferentiate. But other injured cells may irreversibly enter an aberrant state that induces a proinflammatory phenotype, attracting and activating additional immune cells from the circulation and stimulating fibrosis (17, 67). This aberrant state may persist for months to years. A full understanding of events that determine whether a cell will undergo adaptive versus maladaptive repair is lacking, although the latter is enhanced when the injury is more severe (69).

The evidence presented here suggests that the hibernating 13-LGS is an ideal model with which to identify the pathways that determine maladaptive versus adaptive repair. The earliest response to IRI injury, i.e., activation of IER genes, occurred as the animals rewarmed from torpor, but the tempo of downstream events differed, as well as the final outcome, as the hibernators showed no histopathological evidence of enhanced cell death, widespread immune activation, or maladaptive repair, consistent with earlier findings (Supplemental Table S2; 8, 13). Yet the outlier SA animal, K136, proves that this is not a species-specific limitation, because it showed all the classical gene expression signatures of the maladaptive repair pathway (Supplemental Fig. S3). Because the hibernator repeatedly undergoes IRI during rewarming from torpor, any injury incurred must be repaired before Ent or the damage would accumulate over the hibernation season. We found little evidence of immune stimulation; only the two markers for kidney-resident macrophages and one for B-cells were detected across all of the samples (Supplemental Fig. S3) during normal hibernation. Functional enrichment annotations of coexpressed genes (Fig. 3, Supplemental Table S4) in addition to the comparisons with mouse IRI data (Fig. 4, Table 2) show that genes related to cell death and immune responses were quickly but transiently activated only in IBA, in sharp contrast to mouse, where both extend for over a week. A modest increase in caspase 3 activity during IBA compared with torpor and SA was reported previously (13), consistent with our RNA-Seq findings. We speculate that kidney-resident macrophages eliminate any injured cells during IBA by apoptosis, but the signals that recruit circulating immune cells are never sent. The seasonally decreased metabolic genes, also a characteristic of the response to IRI in mice, in addition to the seasonally increased cell division and DNA replication and repair genes, would provide an enhanced background that enables rapid tissue repair via the adaptive pathway in these hibernators. The balance between cell survival and cell death pathways generally favors survival but, in a small number of individual cells, particularly in IBA, this balance can likely change to promote apoptosis, consistent with the assignment of *Bak1*, *Bax*, and *Bcl2l11* to the IBA_high co-expression cluster (Supplemental Fig. S6). Single-cell data are required to understand all of these gene expression changes in their cell-specific context.

To summarize, our histopathological analysis shows that 13-LGS avoids the kidney’s maladaptive repair pathway throughout many months of hibernation, a finding corroborated by the RNA-Seq data. In normal hibernation, the animals rapidly and efficiently execute the adaptive repair pathway during each arousal from torpor. Activation of IER genes during rewarming from torpor occurred in a cellular context seasonally primed for rapid repair by upregulated cell division and DNA repair and replication genes (Winter_high genes) and downregulated (Winter_low) genes involved in mature kidney epithelial transport functions. This seasonal reprogramming may be cued by the metabolic changes associated with fasting, as these animals stop eating in preparation for hibernation and fast throughout winter hibernation (9). Importantly, genes associated with damage and repair, including those related to cell death and the immune system, were activated during IBA, but only transiently, when pathways related to gene expression and proteostasis were also enhanced. By the time the hibernating 13-LGS reentered torpor, repair was complete, with genes exhibiting increased expression being dominated by the mature differentiated kidney features of cilia and mitochondria. The gene expression program that unfolded across the 12 h arousal period in hibernating 13-LGS is highly reminiscent of the 12-h “circatidal rhythm” (Fig. 6), initially observed in animals of the intratidal zone, but now known in mammals (57, 73–75). The important cue for initiating this program is likely rewarming, with its induction of the IER genes. The transcription factor, Xbp1, plays a major role in orchestrating this rhythm (57). Xbp1 is a key component of the unfolded protein response, an IER gene (76), and critical for avoiding maladaptive repair after AKI (77). The functional enrichments in our coexpression clusters, as well as those in the 13-LGS kidney genes that overlap with known 12-h rhythm genes in mouse liver (Fig. 6), indicate that hibernators exploit the 12-h rhythm during arousal, but more frequent sampling across the arousal is needed for confirmation (58). If synchronous activation of this ancient rhythm is necessary and sufficient to ensure adaptive repair of the kidney in hibernators, it may lead to safe, novel treatments that prevent AKI to CKD progression and improve transplant outcomes.

## Data Availability

Raw FASTQ files and batch-corrected, regularized log transformed counts for each library are available in the GEO record, GSE227101.

## SUPPLEMENTAL MATERIAL

Supplemental Figs. S1–S6 and Supplemental Tables S1–S4: https://doi.org/10.6084/m9.figshare.32319390.
